# Integrative proteomic profiling of lung tissues and blood in acute respiratory distress syndrome

**DOI:** 10.3389/fimmu.2023.1158951

**Published:** 2023-05-01

**Authors:** Rui Gong, Hong Luo, Gangyu Long, Jiqian Xu, Chaolin Huang, Xin Zhou, You Shang, Dingyu Zhang

**Affiliations:** ^1^ The First Affiliated Hospital of USTC, Division of Life Sciences and Medicine, University of Science and Technology of China (USTC), Hefei, Anhui, China; ^2^ Center for Translational Medicine, Wuhan Jinyintan Hospital, Tongji Medical College, Huazhong University of Science and Technology (HUST), Wuhan, Hubei, China; ^3^ Department of Critical Care Medicine, Union Hospital, Tongji Medical College, Huazhong University of Science and Technology (HUST), Wuhan, Hubei, China; ^4^ SpecAlly Life Technology Co., Ltd, Wuhan, Hubei, China

**Keywords:** biomarkers, acute lung injury, acute respiratory distress syndrome, proteomics, parallel reaction monitoring, differentially expressed proteins

## Abstract

**Introduction:**

Acute respiratory distress syndrome and acute lung injury (ARDS/ALI) still lack a recognized diagnostic test and pharmacologic treatments that target the underlying pathology.

**Methods:**

To explore the sensitive non-invasive biomarkers associated with pathological changes in the lung of direct ARDS/ALI, we performed an integrative proteomic analysis of lung and blood samples from lipopolysaccharide (LPS)-induced ARDS mice and COVID-19-related ARDS patients. The common differentially expressed proteins (DEPs) were identified based on combined proteomic analysis of serum and lung samples in direct ARDS mice model. The clinical value of the common DEPs was validated in lung and plasma proteomics in cases of COVID-19-related ARDS.

**Results:**

We identified 368 DEPs in serum and 504 in lung samples from LPS-induced ARDS mice. Gene ontology (GO) classification and Kyoto Encyclopedia of Genes and Genomes (KEGG) analysis showed that these DEPs in lung tissues were primarily enriched in pathways, including IL-17 and B cell receptor signaling pathways, and the response to stimuli. In contrast, DEPs in the serum were mostly involved in metabolic pathways and cellular processes. Through network analysis of protein-protein interactions (PPI), we identified diverse clusters of DEPs in the lung and serum samples. We further identified 50 commonly upregulated and 10 commonly downregulated DEPs in the lung and serum samples. Internal validation with a parallel-reacted monitor (PRM) and external validation in the Gene Expression Omnibus (GEO) datasets further showed these confirmed DEPs. We then validated these proteins in the proteomics of patients with ARDS and identified six proteins (HP, LTA4H, S100A9, SAA1, SAA2, and SERPINA3) with good clinical diagnostic and prognostic value.

**Discussion:**

These proteins can be viewed as sensitive and non-invasive biomarkers associated with lung pathological changes in the blood and could potentially serve as targets for the early detection and treatment of direct ARDS especially in hyperinflammatory subphenotype.

## 1 Introduction

ARDS/ALI is a major manifestation of multiple organ failure, characterized by progressive hypoxemia and respiratory distress. It is a not uncommon disorder in intensive care units (ICU), representing 10.4% of ICU admissions, and the mortality rate is high, approximately 34.9%-46.1% (1). ARDS, a heterogeneous syndrome with various underlying pathologies, is generally caused by ‘direct’ lung injury (e.g., pneumonia, aspiration) or ‘indirect’ lung injury (e.g., sepsis, pancreatitis) (2). Clinically, ARDS is defined by the acute onset of bilateral lung infiltrates and hypoxaemia (a low ratio of partial pressure of arterial oxygen normalized to the fraction of inspired oxygen [Pa O_2_/FI O_2_]≤ 300) absent isolated hydrostatic edema typical of congestive heart failure (3). The present definition, although pragmatic, is based on aspecific clinical criteria to categorize grouping patients with vastly different pathophysiologies and outcomes. Recent studies have shown that ARDS can be divided into two subtypes: “hyperinflammatory” and “hypoinflammatory” subphenotype, based on clinical features such as oxygenation index and inflammatory biomarkers such as the levels of IL-6, IL-8, TNF-alpha, and ACE2 (4, 5). The hyperinflammatory subtype of ARDS is usually associated with a poor prognosis and a high mortality rate (4). Therefore, there is an instant need for biomarkers that can serve as predictors for timely diagnosis and intervention of ARDS/ALI, especially hyperinflammatory subtype of ARDS during the ongoing COVID-19 pandemic.

Considering that every physiological activity stems from proteins, research in the field of proteomics has the potential to uncover fundamental mechanisms of biological activities, including pivotal roles in pathophysiologic phenomena, thus offering new possibilities for identification of disease biomarkers. Recently, a quantitative proteomics approach based on data-independent acquisition (DIA) that employs mass spectrometry has been recognized as a major advancement in protein quantification and is significant because of its superior accuracy and reproducibility (6, 7). The parallel-reacted monitor (PRM) method was developed as a means of targeted mass spectrometry quantification with a high degree of precision and resolution. In recent years, the integration of untargeted DIA and targeted PRM methodologies has shown significant potential for identifying and verifying prognostic and predictive biomarkers for diverse diseases, including cancer, liver fibrosis, and cerebral ischemia-reperfusion injuries (8–10).

The intratracheal LPS-induced ARDS model is a classic model of direct factor-induced lung injury (11), a hyperinflammatory phenotype of ARDS (12–14), which results in progressive hypoxemia and respiratory distress. Our study is the first – to the best of our knowledge– to integrate DIA and PRM methods to detect biomarkers related to lung pathological changes in hyperinflammatory subtype of ARDS in mice. Subsequently, we validated the results using external datasets from the GEO database and previous proteomics outcomes from our study of patients with COVID-19-induced ARDS (15, 16). From the integrative proteomic analysis, we identified six sensitive non-invasive biomarkers with high clinical prognostic value in the blood that are associated with pathological changes in the lungs, providing potential targets for the early detection and treatment of ARDS, especially the hyperinflammatory subtype.

## 2 Materials and methods

### 2.1 Animal models

Male C57BL/6 mice aged 8-10 weeks were procured from Si Pei Fu Biotech Co., Ltd. (Beijing, China). In a normally maintained facility, all mice were housed at 22°C with a 12-h light cycle and were given free access to food and drink. All procedures were conducted in accordance with the guidelines of Chinese Animal Research and were approved by the Experimental Animal Ethics Committee of Wuhan Xianran Biotechnology Co., Ltd. (Hubei, China).

Thirty mice were randomly assigned to control (n = 15) and experimental ARDS (n = 15) groups. Mice in the experimental group were intratracheally injected with 5 mg/kg LPS (Sigma) to establish the ARDS/ALI model, and the counterparts in control group were received the corresponding volume of PBS. The mice were sacrificed 24 h after LPS challenge, and blood, bronchoalveolar lavage fluid (BALF), and lung samples were obtained.

### 2.2 Evaluation of lung injury

BALF was obtained as previously described (17). Briefly, after the ligation of left lung, the right lung was perfused with 0.5 mL of 4 °C PBS three times, followed by the collection of BALF from the right lung. Subsequently, BALF samples were centrifuged at 500 g and 4 °C for 10 min. The supernatants were harvested for total protein concentration assay, and cell pellets were resuspended in 500 μL PBS for total cell counting. The left lungs of mice were processed for hematoxylin and eosin (HE) staining to assess the degree of lung injury, as reported previously (18), and wet and dry lung weights were measured to acquire the wet/dry ratio of the lung.

### 2.3 DIA proteomics analysis

Blood samples were collected after eyeball enucleation and placed for 1 h at room temperature, after which supernatants were centrifuged at 1,000 g for 10 min at 4°C to acquire serum samples. After the collection of blood from the euthanizes mice, whole lungs were immediately harvested and washed three times with PBS. Serum and lung samples were stored at -80°C for further analysis.

Lung and serum samples from five mice in each group were processed for the DIA proteomics quantification. In brief, the total protein was extracted and measured with BCA method. Protein reduction and alkylation were conducted with TCEP (tris (2-carboxyethyl) phosphine hydrochloride) and CAA (2-chloroacetamide), followed by overnight trypsin digestion (SignalChem). Digested peptides were desalted using a self-prepared SDB-RPS desalting column. Desalted peptides were loaded onto a timsTOF Pro mass spectrometer (Bruker Daltonics) coupled with an UltiMate 3000 RSLC nano-system (Thermo Fisher Scientific) and analyzed in diaPASEF mode. The raw DIA data were searched against the reviewed mouse database from UniProt (2020-08-26, 17053 entries) using DIA-NN software (V1.8.1). Proteins with a |log2 FC| > 0.585 (fold change >3/2 or <2/3) and P < 0.05 (Student’s t-test) were identified as DEPs. Gene ontology (GO) classification and Kyoto Encyclopedia of Genes and Genomes (KEGG) enrichment of DEPs were conducted using the “cluster Profiler” packages (significant at P < 0.05).

### 2.4 Targeted PRM analysis

Lung tissues and serum from three mice in each group were used for targeted PRM analysis. Protein extraction, reduction, alkylation, digestion, and desalting were conducted as described for DIA proteomic analysis. Desalted peptides were loaded into a Q Exactive HF mass spectrometer (Thermo Fisher Scientific) coupled with an UltiMate 3000 RSLC- nano system (Thermo Fisher Scientific) and analyzed with PRM mode. Raw PRM data were analyzed using Skyline software (v22.2.0.312) to extract the peak areas of the target precursors.

### 2.5 Microarray data analysis

The GEO databases GSE2411 and GSE104214 were utilized to perform the analysis of the expression of genes corresponding to the common DEPs in LPS-induced ARDS/ALI. The normalization of raw data was carried out through MicroArray Suite 5.0 (MAS5.0) using the “affy” package.

### 2.6 Construction of the PPI network

The PPI network was analyzed for DEPs using the STRING database. The network nodes were scored using CytoHubba. In addition, we utilized Cytoscape to perform clustering analysis by the molecular complex detection (MCODE) algorithm, in which the criteria were set as belows: degree cut-off = 2, node score cut-off = 0.2, maximum depth = 100, k-score = 2, and high confidence ≥ 0.7.

### 2.7 ROC curves of target proteins in ARDS patients

Based on our group previous proteomic results of ARDS patients caused by COVID-19 (15), ROC curves were created and the area under the ROC curve (AUC) was calculated separately to evaluate the diagnostic performance of the target proteins using the R packages “pROC”.

### 2.8 Serological validation of target proteins in ARDS patients

Different plasma levels of the target proteins were evaluated in serum samples of ARDS patients with different severity using previous proteomic data (15). Data were analyzed by Pairwise test.

### 2.9 Statistical analysis

All statistical analysis was conducted with Prism Graphic software (version 9) using the t-test for the two groups. All analysis was peformed at least three times, and representative experimental results are presented. Significant differences were judged to exist when P < 0.05.

## 3 Results

### 3.1 Establishment of ARDS model in mice

**Figure 1:**
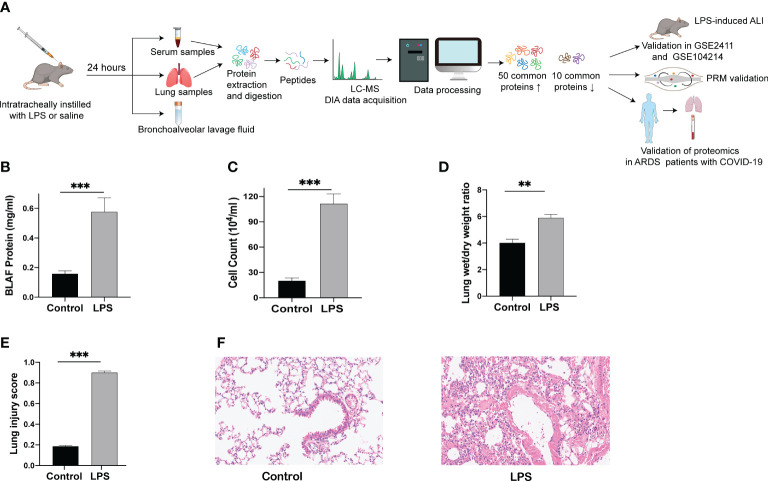
Lung injury induced by intratracheal LPS in mice. **(A)** Schematic diagram of the experimental protocol. **(B)** BALF protein concentration as determined by ELISA. **(C)** Total cell counts in BALF measured using a hemocytometer. **(D)** Wet/dry weight ratio of the lung was computed for assessment of pulmonary edema. **(E, F)** HE staining of lung tissues (200 x) and histopathological score. ** p < 0.01; *** p < 0.001.

The establishment of a human disease-simulating animal model aids in understanding the mechanisms underlying disease occurrence and development. A flow chart depicting the progress of the animal experiments and comprehensive analysis of serum and lung tissue proteomics is shown in **Figure 1A**. After 24 h of LPS challenge, mice in the experimental group exhibited increases in protein levels of BALF, cell counts in BALF, and wet/dry ratio (**Figures 1B-D**), in contrast to the control group. In parallel with the above indicators, the lung tissue morphology evaluated through HE staining indicated a higher lung injury score after the LPS challenge (**Figure 1E**), accompanied by increased inflammatory cell infiltration, alveolar hemorrhage and collapse, and alveolar wall thickness in the experimental group (**Figure 1F**). These results indicate that the mice model of ARDS/ALI was successfully established.

### 3.2 Quality control and verification

The false discovery rate (FDR) value was set to 1% for reliable precursor identification, and proteins with at least 50% valid FDR values in at least one replicate group were used for further analysis. In total, 8,313 proteins and 101,282 precursors were identified by quantitative DIA analysis of all lung samples (**Figure S1A**). The verification of quality control is presented in **Figures S1B-E**, including the length and number distribution of peptides, the distribution of missed tryptic cleavage sites, and the distribution of missing data. As shown in **Figure S1B**, almost all the peptides have a length between 7 and 27, which is in accordance with the tryptic property of the peptides. According to the peptide number distribution shown in **Figure S1C**, the majority of the proteins contained two or more peptides. The missed cleavage sites of most peptides were nearly zero based on the trypsin digestion data (**Figure S1D**). Proteins with missing values in each sample accounted for less than 2.6% of the distribution of missing data (**Figure S1E**). Overall, these results implied that proteomic profiling was highly reliable at the peptide level. Moreover, principal component analysis (PCA) revealed that the protein expression between the control and ARDS samples was clearly separated (**Figure S1F**). Additionally, the results of Pearson’s correlation analysis revealed an obvious difference between the control and ARDS/ALI mice but slight differences within each group, representing good quantitative reproducibility between the two groups (**Figure S1G**). Similarly, the quality control and verification were also completed in the serum samples. As presented in **Figures S2A-G**, the results of the proteomic data in the serum samples also met the criteria of quality control, exhibited distinct separation between two groups and good biological repeatability in each group.

### 3.3 Identification of lung DEPs in ARDS mice and bioinformatic analysis

**Figure 2:**
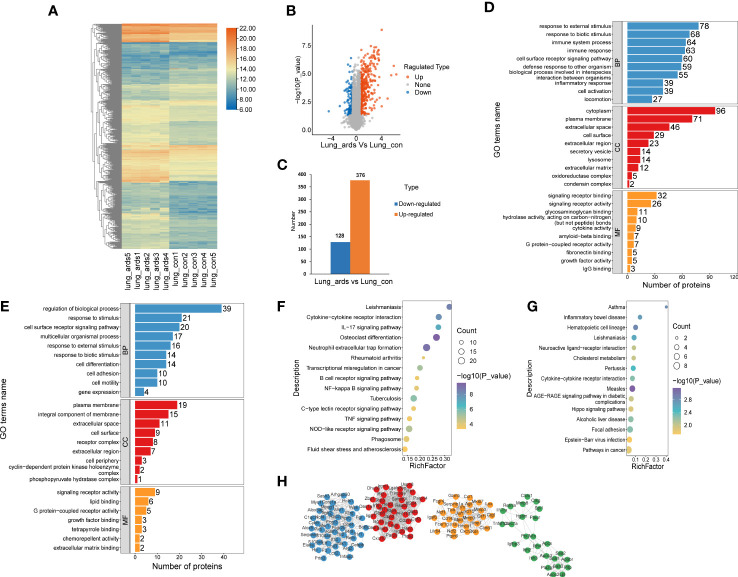
Recognition and bioinformatics analysis of lung DEPs in ARDS mice as compared to controls. Distribution of the lung DEPs in ARDS mice (compared with controls) is shown in **(A)** heatmap, **(B)** volcano, and **(C)** histogram. GO analysis of the upregulated **(D)** and downregulated **(E)** proteins in lung tissues of ARDS mice in contrast to controls. KEGG analysis of upregulated **(F)** and downregulated **(G)** DEPs in mice lung tissues. **(H)** PPI network of the optimized DEPs.

A total of 504 proteins showed prominent differences in the lungs of ARDS mice, including 376 upregulated DEPs and 128 downregulated DEPs. The volcano plots, heat maps, and histograms of the DEPs are presented in **Figures 2A-C**, respectively. GO analysis disclosed that DEPs mainly participated in the following activities: responses to external and biotic stimuli, regulation of biological processes, cell surface receptor signaling pathway (biological process, BP), which originated from the cytoplasm, plasma membrane, extracellular space, and integral component of membrane (cellular component, CC), signaling receptor binding, signaling receptor activity, and lipid binding (molecular function, MF) (**Figures 2D, E**). KEGG enrichment analysis indicated that the upregulated DEPs were mostly enriched in neutrophil extracellular trap formation, osteoclast differentiation, NOD-like receptor signaling pathway, and cytokine-cytokine receptor interaction. The downregulated DEPs were mostly involved in neuroactive ligand−receptor interaction, cytokine−cytokine receptor interaction, and the AGE−RAGE signaling pathway (**Figures 2F, G**).

As shown in **Figure 2H**, we utilized the STRING database to analyze the protein network of 504 DEPs and subsequently employed MCODE to extract the four sub-networks with the highest scores from the revealed 11 clusters. The core proteins of these clusters are as follows: tumor stroma and activated macrophage protein DLM-1, GP90 lymphocyte homing/adhesion receptor, tissue inhibitor of metalloproteinases 1, dendritic cell-associated C-type lectin 1, phospholipase A2 inhibitory protein, hexokinase type II, interleukin-1 receptor-associated kinase 3, mitogen-activated protein kinase p38 delta, V-maf musculoaponeurotic fibrosarcoma oncogene family, protein F (avian), structural maintenance of chromosomes protein 2, and growth differentiation factor 15 (**Supplemental Table 1**).

### 3.4 Identification of serum DEPs in ARDS mice and bioinformatic analysis

**Figure 3:**
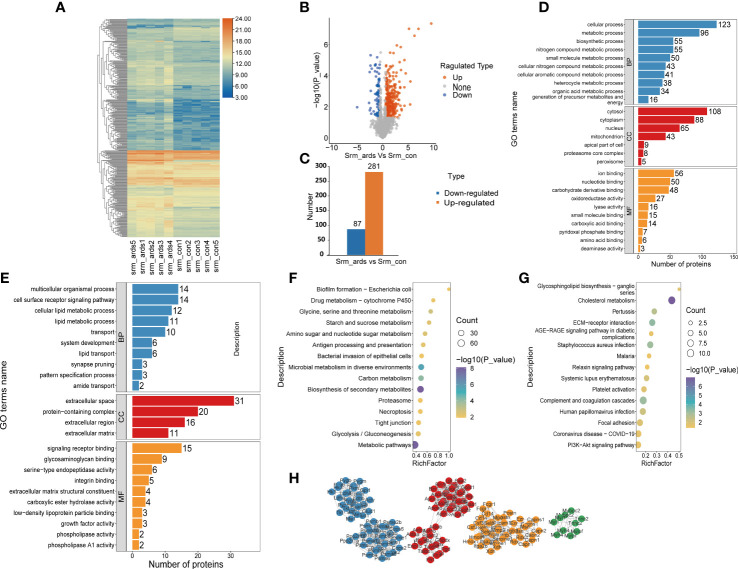
Recognition and bioinformatics analysis of serum DEPs in ARDS mice compared with controls. **(A)** Heatmap, **(B)** volcano, and **(C)** histogram depicting the distribution of the serum DEPs in ARDS mice compared with controls; GO analysis of **(D)** the upregulated proteins and **(E)** downregulated proteins in serum of ARDS mice in contrast to controls; KEGG analysis of upregulated proteins **(F)** and down regulated DEPs **(G)** in mice serum; **(H)** PPI network of the optimized DEPs in serum of mice.

Because alterations in protein expression in the plasma or serum could reflect pathophysiological changes in organs in various human diseases, we also analyzed the serum proteomic data in ARDS/ALI mice. In the serum of ARDS mice, 368 proteins were significantly different, including 281 upregulated DEPs and 87 downregulated DEPs. Volcano plots, heat maps, and histograms of the DEPs are shown in **Figure 3A-C**. As revealed by the GO enrichment analysis, DEPs were mostly involved in cellular processes, metabolic processes, multicellular organismal processes, and cell surface receptor signaling pathways (BP) originating from the cytosol, cytoplasm, protein-containing complex, and extracellular region (CC), and were engaged in ion binding, nucleotide binding, signaling receptor binding, and glycosaminoglycan binding (MF) (**Figures 3D, E**). KEGG analysis illustrated that the upregulated DEPs were mainly enriched in drug metabolism; glycine, serine, and threonine metabolism; and starch and sucrose metabolism. In contrast, the downregulated DEPs were mostly involved in glycosphingolipid biosynthesis, cholesterol metabolism, and interactions of the ECM and receptors (**Figures 3F, G**).

As illustrated in **Figure 3H**, we employed the STRING database to analyze the protein network of 368 DEPs, followed by MCODE to extract the top four subnetworks from the 11 clusters. The core proteins identified were: succinate dehydrogenase [ubiquinone] flavoprotein subunit, mitochondrial; apolipoprotein C-III; phosphoglycerate mutase isozyme B; protein Z, vitamin K-dependent plasma glycoprotein; radixin; ARP3 actin-related protein 3; tumor metastatic process-associated protein; RAB11B, member RAS oncogene family; myosin light chain, phosphorylatable, fast skeletal muscle; complement component 1, q subcomponent, beta polypeptide; inosine-guanosine phosphorylase; serine/threonine-protein phosphatase 2A catalytic subunit alpha isoform; and scavenger receptor cysteine-rich type 1 protein M130 (**Supplemental Table 2**).

### 3.5 Common DEPs in the lungs and serum samples of ARDS mice

**Figure 4:**
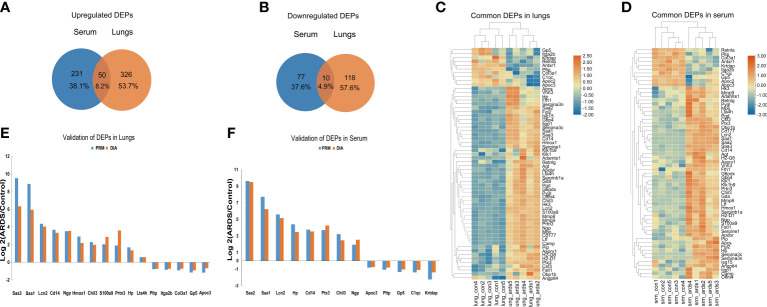
Common DEPs in the serum and lungs of ARDS mice. **(A)** Common DEPs upregulated in the serum and lungs of ARDS mice (n = 5). **(B)** Common DEPs downregulated in the serum and lungs of ARDS mice (n = 5). **(C, D)** Heatmap of the common DEPs in lung and serum samples. **(E, F)** PRM validation of common DEPs in lung and serum samples.

Upon examining the intersection of DEPs in the lungs and serum to identify common DEPs with the same expression trends, we found 50 commonly upregulated and 10 downregulated DEPs in the serum and lung samples (**Figures 4A, B**; **Table 1**, **2**). In **Figures 4C, D**, we show the heat maps of common DEPs in the serum and lung samples.

**Table 1:** 50 commonly upregulated DEPs in both lung tissue and serum of ARDS/ALI mice compared to controls.

Accession	Protein	Log2 fold change (lung)	Log2 fold change (serum)
P04918	Saa3	6.342378258	7.0214628
P05366	Saa1	5.94415259	6.237169
P05367	Saa2	5.545448168	9.5184333
P11672	Lcn2	4.03902095	5.1579087
P48759	Ptx3	3.649367946	4.2621869
Q61096	Prtn3	3.600477415	3.3194135
O08692	Ngp	3.547450731	2.5409037
Q8R2S8	Cd177	3.516108839	2.1243307
P51437	Camp	3.345238261	1.3489157
P08071	Ltf	3.345018494	2.1092754
P10810	Cd14	3.332715545	3.5366451
P11247	Mpo	3.147273468	1.4498862
P22777	Serpine1	2.971851335	3.4348432
Q3TRM8	Hk3	2.892972242	1.4145415
P31725	S100a9	2.869175648	1.2725338
P41245	Mmp9	2.755070104	1.472634
Q91WP6	Serpina3n	2.561340179	2.0043568
O70138	Mmp8	2.507102878	1.7046071
Q3UZZ4	Olfm4	2.484007335	1.5571941
P12246	Apcs	2.453424421	2.3012683
P15947	Klk1	2.447389196	2.5177009
Q09PK2	Asprv1	2.248596895	1.1805633
P14901	Hmox1	2.190951681	3.2061107
O35744	Chil3	1.98494947	2.4727863
P15949	Klk1b9	1.974060884	4.2533979
Q8K426	Retnlg	1.724696691	1.0714405
P11859	Agt	1.666590677	1.46822
P29621	Serpina3c	1.610342656	1.1621107
Q9QZ85	Iigp1	1.540575384	1.1493861
Q9Z1P8	Angptl4	1.442310789	2.1256101
P09920	Csf3	1.433805889	5.4357411
Q61646	Hp	1.357568005	3.4814159
A2AEP0	Obp1b	1.311576901	2.7214961
Q61107	Gbp4	1.290745458	1.9415751
Q8VBT6	Apobr	1.279600792	2.3378003
P14426	H2-D1	1.205855276	1.3711146
Q9ET01	Pygl	1.204219638	0.9719964
Q9D154	Serpinb1a	1.113847575	0.6237937
P02816	Pip	1.092258191	1.824279
P14430	H2-Q8	1.068915367	1.224011
P97857	Adamts1	0.897208004	1.4199632
P12804	Fgl2	0.895568168	1.2289563
Q9QZ25	Vnn3	0.864197348	2.8269183
Q00612	G6pdx	0.856947747	0.990037
Q62356	Fstl1	0.692738016	0.8296838
Q64339	Isg15	0.673438366	0.8737584
P09528	Fth1	0.62918251	2.3243612
Q9R111	Gda	0.606739355	1.3476802
Q9DCD0	Pgd	0.589503503	1.8115959
P24527	Lta4h	0.588354947	1.5900244

**Table 2:** 10 commonly downregulated DEPs in both lung tissue and serum of ARDS/ALI mice compared to controls.

Accession	Protein	Log2 fold change (lung)	Log2 fold change (serum)
Q9EP95	Retnla	-2.601009556	-1.428189185
Q9CZ52	Antxr1	-1.303873136	-0.833925223
Q3V2T4	Krtdap	-1.105530289	-1.378035745
Q05020	Apoc2	-0.929274914	-1.472628626
O08742	Gp5	-0.903588651	-1.037650735
P55065	Pltp	-0.740646594	-0.796303175
Q9QUM0	Itga2b	-0.736701388	-0.652713319
P08121	Col3a1	-0.721446883	-2.552194434
P33622	Apoc3	-0.660992733	-0.743100519
Q02105	C1qc	-0.644895958	-1.085356984

PRM is an ion-monitoring method that employs high-resolution, high-precision mass spectrometry, in which target proteins and peptides can be detected in terms of both relative and absolute quantification. To verify the common DEPs from DIA proteomic quantification in the serum and lung samples, we randomly selected 13 DEPs to perform further PRM validations (**Supplemental Tables 3, 4**). As expected, the trend of common DEPs evident in the PRM results was essentially the same as what was found in the proteomic analysis, confirming the reliability of our proteomic data through internal validation (**Figures 4E, F**).

### 3.6 External validation of common DEPs

**Figure 5:**
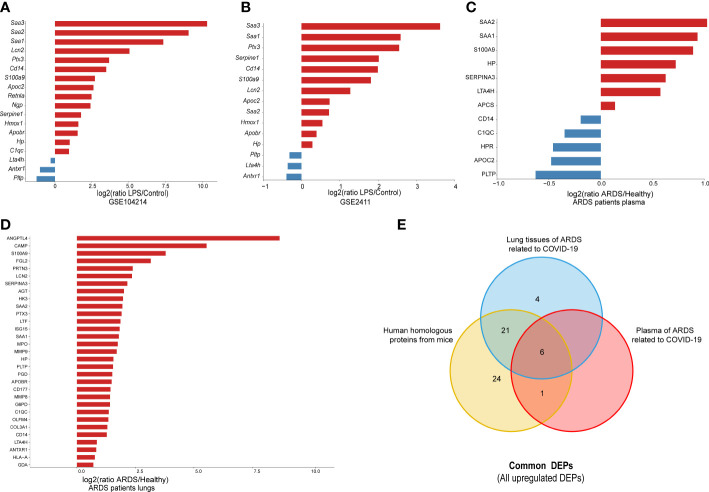
The external validation of common DEPs, using different datasets: **(A)** 20 randomly selected common DEPs in GSE104214 (p < 0.05); **(B)** 20 selected common DEPs in GSE2411 (p < 0.05); **(C)** human orthologs in plasma proteomics analysis of ARDS cases caused by COVID-19 (p < 0.05); and **(D)** human orthologs in lung proteomics analysis of ARDS cases caused by COVID-19 (p < 0.05); **(E)** Venn diagram for all common DEPs among proteins from lung and plasma of ARDS patients, and human homologous proteins from mice.

To further confirm the validity of the identified DEPs, we utilized GEO datasets (GSE104214 and GSE2411) in which transcriptome sequencing of lung tissue was performed in LPS-induced ALI mice. We performed computational validation of 20 randomly selected common DEPs at the mRNA level and found 18 genes encoding common DEPs exhibited significantly different expression in GSE 104214, in which the upregulated genes were as follows: Serum Amyloid A3 (Saa3), Serum Amyloid A1 (Saa1), Pentraxin-Related Protein PTX3 (Ptx3), Plasminogen Activator Inhibitor 1 (Serpine1), Monocyte Differentiation Antigen CD14 (Cd14), Protein S100-A9 (S100a9), Neutrophil Gelatinase-Associated Lipocalin (Lcn2), Apolipoprotein C-II (Apoc2), Serum Amyloid A2 (Saa2), Heme Oxygenase 1 (Hmox1), Apolipoprotein B Receptor (Apobr), Haptoglobin (Hp), Resistin-Like Alpha (Retnla), Neutrophilic Granule Protein (Ngp), Complement Component 1, q Subcomponent, and C Chain (C1qc). The downregulated genes were Phospholipid Transfer Protein (Pltp), Leukotriene A4 Hydrolase (Lta4h), and Anthrax Toxin Receptor 1(Antxr1) (**Figure 5A**).

Similarly, the 15 genes that showed significant differences in GSE 2411 were mostly consistent with the above results, except for three upregulated genes, Retnla, Ngp, and C1qc (**Figure 5B**). Collectively, the results of the external validation confirmed that the proteomic data were highly reliable.

Subsequently, to further explore the clinical value of these common DEPs in patients with ARDS, we identified 60 common DEPs in cases of ARDS caused by COVID-19, which is currently the most common direct cause of lung injury. Mouse genes from the above 60 DEPs were mapped to 62 human homologs identified based on the MGI database(52 upregulated and 10 downregulated proteins). Based on our previous proteomic data of lung and plasma samples from patients with ARDS induced by COVID-19 (15, 16), we identified 31 human homologous proteins with distinct expression changes in lung tissues and 12 homologous proteins with significant expression differences in plasma samples among the 62 homologous proteins (**Figures 5C, D**). A dataset was constructed from a) the intersection of 52 upregulated homologous proteins originating from common DEPs in mice and b) the upregulated proteomic results of lung and plasma samples from ARDS patients. From this dataset, six target DEPs, namely HP, LTA4H, S100A9, SAA1, SAA2, and SERPINA3, were identified with co-upregulated trend in lung and blood (**Figure 5E**). While, no commonly downregulated DEPs were identified in the intersection of DEPs of downregulated groups.

### 3.7 Clinical value assessment of common DEPs

**Figure 6:**
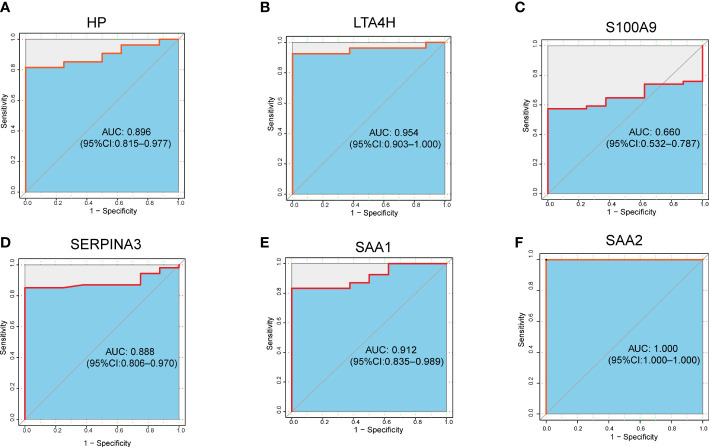
ROC analysis of 6 target DEPs in plasma proteomics analysis of ARDS cases with COVID-19. **(A)** HP; **(B)** LTA4H; **(C)** S100A9; **(D)** SERPINA3; **(E)** SAA1; **(F)** SAA2.

To further evaluate the clinical value of the six target DEPs, we generated receiver operating characteristic (ROC) curves based on the aforementioned proteomics data from the plasma of ARDS patients with COVID-19 (including 5 nonsurvivors, 7 severe cases, 10 mild cases, and 8 healthy subjects) (10). The AUC values of six proteins we proposed as potential biomarkers, are ranging from 0.660 to 1.000, with more than half (5 proteins) showing an AUC greater than 0.8 (**Figures 6A-F**).

**Figure 7:**
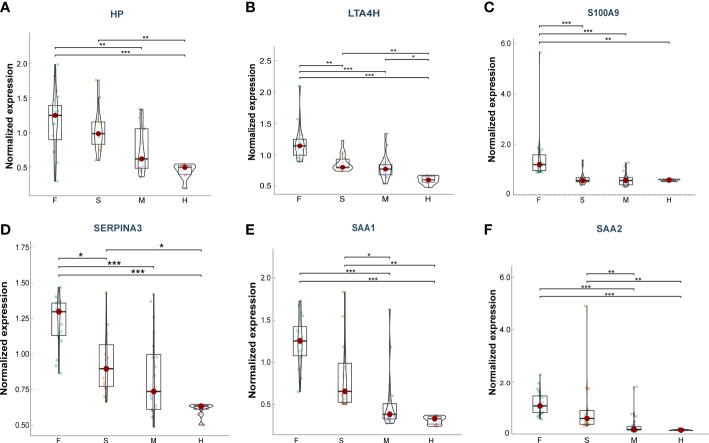
Plasma levels of the target proteins from the serum samples of ARDS patients caused by COVID-19. **(A)** HP; **(B)** LTA4H; **(C)** S100A9; **(D)** SERPINA3; **(E)** SAA1; **(F)** SAA2. Data points are presented for each individual patient, shown as median values with interquartile ranges (F, n = 20; S, n = 14; M, n = 20; H, n = 8). The center line of each box indicates the median and the top and bottom of the box represent the 75th and 25th percentile values, respectively. * p < 0.05; ** p < 0.01; *** p < 0.001.

Furthermore, we explore the prognostic value of these target proteins in ARDS patients with different outcomes (Fatal (F) = 20, Severe (S) = 14, Mild (M) = 20, Healthy (H) =8) (15). We found that the plasma levels of HP, LTA4H, S100A9, SAA1, SAA2, and SERPINA3 were significantly elevated in more severe COVID-19 conditions (**Figures 7A–F**), exhibiting good clinical predictive value and promising applications.

## 4 Discussion

ARDS/ALI is a life-threatening condition with heterogeneous etiologies that poses a great challenge to public health worldwide, particularly during the COVID-19 pandemic. According to the pathophysiological mechanism of ARDS, it can be categorized into direct and indirect lung injury (2), during which direct lung injury (direct ARDS) shares a similar pathological damage characterized by more severe lung epithelial injury and subsequent loss of the alveolar–capillary barrier function, that leads to an influx of protein-rich edema fluid (19, 20). Additonally, there are two distinct subphenotypes: “hyperinflammatory” and “hypoinflammatory” subtype, in direct ARDS. Moreover, a growing body of evidence suggest the hyperinflammatory subtype of ARDS is usually associated with a poor prognosis and a high mortality rate (21–23). Therefor, It is of great importance to early idenyify hyperinflammatory subphenotype of ARDS, which has crucial clinical implications for reducing mortality in ARDS patients.

In this study, we performed a proteomic analysis of protein alterations in lung and serum samples of ARDS/ALI mice induced by intratracheal LPS, the classical hyperinflammatory model of direct lung injury leading to ARDS, to explore non-invasive biomarker panels reflecting lung pathological changes in the blood. Our proteomic analysis identified 504 DEPs from the lung tissues that were mainly involved in IL-17 and B cell receptor signaling pathways response to stimuli, immune system process, and cell surface receptor signaling pathway. In addition, the 368 DEPs in the serum samples were mostly involved in metabolic pathways and participated in cellular, metabolic, and biosynthetic processes. Integrative analysis of the above proteomic results identified 60 common DEPs, of which 50 were upregulated and 10 were downregulated. Thirteen DEPs were randomly selected for internal validation using PRM, and the same trend of DEPs was seen in the quantitative PRM results as was found in the proteomic analysis. Moreover, we validated the expression of 20 randomly selected common DEPs at the mRNA level in two external GEO datasets that showed the same trend of alterations. Subsequently, to further determine the clinical value of these DEPs, we combined 62 human homologous proteins originating from 60 common DEPs of mice with proteomic data from lung tissue and plasma of COVID-19-related ARDS cases and ascertained six proteins of human orthologs (HP, LTA4H, S100A9, SAA1, SAA2, and SERPINA3) with good clinical diagnostic and prognostic value. ROC analysis for these proteins in the plasma proteomics of COVID-19 patients showed high accuracy, with AUC values ranging from 0.660 to 1.000, more than half of which (5 proteins) showed an AUC above 0.8. Additionally, these proteins were shown to be a good indicator of the prognosis of ARDS patients with varying levels of severity, revealing six proteins with the possibility to become promising targets for early detection and prognosis of ARDS.

Haptoglobin (HP), a plasma protein primarily produced by the liver, can capture and combine with free Hb in plasma to promote the recycling of heme iron in liver. HP also functions as an antioxidant, exhibits the properties of antibacteria and modulation of the early acute-phase response following tissue damage (24). Recent studies of HP as an acute-phase reactant in sepsis have focused on the possible relationship between HP and the inflammatory response in sepsis, which is yet to be elucidated (25–27). In a retrospective cohort study of critically septic patients, after adjusting for confounding factors, elevated plasma HP levels were correlated with a decreased risk of in-hospital mortality (28). Erythrocytes are susceptible to damage in sepsis. When these erythrocytes are lysed, hemoglobin is released and oxidized, resulting in infection and an excessive immune response, which together cause tissue damage and organ dysfunction (29). However, HP, an endogenous scavenger of cell-free hemoglobin, may improve sepsis outcomes. Studies of HP supplementation in animal models and associated improved outcomes further support cell-free hemoglobin as a significant contributor to sepsis-related morbidity and mortality (29–31). Moreover, the intraoperative administration of HP in patients undergoing cardiac surgery was independently related with a decreased incidence of AKI following cardiovascular surgery (32). In the present study, we found that HP exhibited significantly increased levels in animal models of ARDS/ALI induced by LPS, which is consistent with the result from our analysis of the plasma samples from patients with COVID-19-induced ARDS, indicating that HP may be a potential marker for predicting the progression of ARDS. Collectively, these results demonstrate that HP plays an important role in lung injury development, warranting further studies to confirm its predictive and therapeutic roles in ARDS of different etiologies.

Leukotriene A4 Hydrolase (LTA4H) is a zinc metalloenzyme with two distinct functions: hydrolase and aminopeptidase. It participates in the final stage of the biosynthetic pathway of leukotriene B4 (LTB4), a classical chemoattractant and immune-modulating lipid mediator, and the degradation of proline-glycine-proline (PGP), a tripeptide that is specifically chemotactic for neutrophils *in vitro* and vivo (33). LTB4 interacts with its high-affinity receptor, BLT1, which is generated by immune cells (such as monocytes, neutrophils, and eosinophils) to recruit and activate other immune cells, particularly neutrophils, during the onset of inflammation (33). In the pathogenesis of COPD, LTA4H released from lung epithelial cells comprises pro-inflammatory and anti-inflammatory properties, which is more inclined to exhibit a pro-inflammatory phenotype (33). To date, modulators of LTA4H have shown the potential to alleviate acute lung injury in animal studies. Xiao et al. found that bufexamac suppressed neutrophil chemotaxis and ameliorated lung injury by directly inhibiting the LTA4H enzymatic reaction, resulting in reduced LTB4 levels in LPS-induced ALI (34). Recently, an Lta4h modulator, 4-methoxydiphenylmethane (4MDM), selectively augmented LTA4H aminopeptidase activity and reduced the wet-to-dry lung weight ratio and degree of leukocyte infiltration in peri-bronchial areas, independent of PGP (35). We found that LTA4H was upregulated in our proteomic analysis of lung and plasma samples and revealed a high AUC value (0.954). Available evidence suggests LTA4H may be an extremely promising biomarker and therapeutic target for ARDS/ALI.

S100 Calcium Binding Protein A9 (S100A9) is a calcium and zinc-binding protein that often exists in the form of the heterodimer S100A8/A9, primarily released by immune cells like neutrophils and macrophages, which participates in various biological functions inside and outside the cell (36). Cytosolic S100A8/A9 complexes play roles in cytoskeletal regulation, arachidonic acid metabolism, and pathogen defense. Extracellular S100A8/A9, in addition to stimulating leukocyte recruitment and cytokine secretion, has anti-inflammatory characteristics under certain conditions, implying that these proteins make a contribution to inflammation homeostasis.

Researchers have shown that S100A9 −/− mice were resistant to E. coli-induced abdominal sepsis (37) and endotoxin-induced cardiomyocyte dysfunction, which suggests that S100A8/S100A9 promotes organ damage by amplifying LPS-induced responses *via* TLR4 (38). The inhibition of S100A9 prevents lung injury induced by intratracheal LPS through blocking the NLRP3 pathway (39). In addition, a cohort study demonstrated that the level of S100A9 mRNA in the blood increased in the progression of sepsis, and its postponed upregulation was linked to the incidence of secondary hospital-acquired infections (40). Hiroshima et al. found that the inhalation of S100A9 and S100A8/A9 reduced neutrophil numbers in BALF and alleviated lung injury in LPS-induced ARDS/ALI models, which may be explained by the restoration of SIRT1 levels to anti-inflammatory functions (41). Our integrative proteomic analysis revealed elevated levels of S100A9 in the blood and lung tissues of mice and patients with ARDS, indicating promising prospects for clinical application. Although clinical trial data on S100A9 are still lacking, S100A9 will likely continue to attract interest as a potential therapeutic target in ARDS/ALI.

Serum amyloid A1 and 2 proteins (SAA1 and SAA2) comprise closely related and highly conserved serum molecules, encode nearly identical proteins that are secreted prominently by hepatocytes and macrophages, and are highly expressed in response to inflammation and tissue injury (42). Apart from partitioning into the HDL fraction in blood and cholesterol transport and recycling, SAA1/2 are also the most conspicuous components of the acute phase response (APR), in which serum levels of SAA1/2 surge sharply in response to trauma, infection, and other stimuli (43). Linke et al. illustrated that SAA treatment improves recovery from severe polymicrobial sepsis in mice, whereas blocking the peptide of the SAA protein results in early death (44). Moreover, Cheng et al. revealed that acute-phase SAA1 offers innate feedback protection against immune response and lung damage induced by LPS and cecal ligation and puncture (CLP) by directly interacting with LPS to generate a complex that enhances LPS clearance in macrophages (45). However, the relationship between SAA1/2 and ARDS/ALI remains unclear. Our proteomic studies demonstrated that the levels of SAA1 and SAA2 were raised in the lungs and blood samples of mice and ARDS patients. ROC analysis showed ideal AUC values for SAA1 (0.912) and SAA2 (1.000) in patients with COVID-19-induced ARDS. Based on the current findings, SAA1/2 can reflect the level of acute inflammation and are expected to be important biomarkers and therapeutic targets for ARDS/ALI.

SERPINA3 (Serpin family A member 3), also called alpha-1 antichymotrypsin, belongs to the serine protease inhibitor (serpin) superfamily of proteins. SERPINA3, which is predominantly produced in the liver and bronchial epithelial cells (46), inhibits the activity of several serine proteases, such as pancreatic chymotrypsin, neutrophil cathepsin G, and mast cell chymases (47). SERPINA3 has the strongest association with cathepsin G, and excessive or prolonged cathepsin G activity caused by inadequate serpin regulation can lead to tissue damage (48, 49). Given that

SERPINA3 is mainly involved in the acute phase response, inflammation, and proteolysis, it could be utilized as a biomarker for the prognosis and diagnosis of several diseases, including myocardial infarction, various cancers, and neurodegenerative diseases (49). Recently, it has been reported that the levels of glycopeptides and glycosylation remodeling associated with SERPINA3 increase substantially during the development of sepsis and sepsis-induced ALI (50, 51). Moreover, Wang et al. reported that SERPINA3 is a potential therapeutic target for LPS-induced ALI (52). SERPINA3 may be an effective inhibitor of pancreatitis-induced lung injury (53). During the COVID-19 pandemic, several proteomics studies have shown increased SERPINA3 levels in COVID-19 related ARDS patients in contrast to healthy controls (54–56), which accords with our earlier findings (15). In addition, our proteomic analysis showed that SERPINA3 levels were significantly increased in the blood and lung tissues of ARDS mice, exhibiting prospective clinical value. However, to date, no studies have elucidated the pathological mechanisms of SERPINA3 in ARDS/ALI of different etiologies. SERPINA3, an inhibitor of neutrophil cathepsin G and mast cell chymase that can transform inactive angiotensin-1 into active angiotensin-2, deserves further study in ARDS/ALI associated with both direct and indirect lung injury factors.

Previous ARDS proteomics studies have predominantly focused on plasma or bronchoalveolar lavage (BAL) fluid, which is relatively easy to obtain and readily available in many large cohorts, but may poorly reflect the pathological changes in the lung (57–59). In our study, we firstly integrate proteomic analysis of serum and corresponding lung samples followed by PRM validation, identifying six blood biomarkers associated with lung pathological changes. However, lung tissues from ARDS patients are more difficult to obtain than serum and plasma samples, limiting their clinical application. We further validated the expression of these six DEPs in lung samples of direct ARDS patients caaused by COVID-19. Furthermore, we found that the expression levels of six DEPs in plasma were correlated positively with the severity of the disease and exhibited good diagnostic value, which provided valuable predictive and prognostic information for both clinicians and investigators.

There are a few inherent limitations in our study. Despite the identified six DEPs showed good clinical value in direct ARDS caused by COVID-19, their clinical value in direct ARDS of other etiologies is uncertain. Meanwhile, the dynamics of these DEPs over time in ARDS progression are not completely elucidated, however early detection of changes in these proteins is more valuable for early diagnosis and treatment of direct ARDS. Moreover, these proteins may contribute to the discovery of hyperinflammatory subtypes of direct ARDS, but the expression of these proteins in hypoinflammatory subtypes requires further validation.

## 5 Conclusions

In this study, we utilized proteomic analysis of data from ARDS cases with COVID-19 as well as an LPS-induced mouse model of ARDS to identify common DEPs that could serve as potential biomarkers in direct ARDS of hyperinflammatory subtype. Six sensitive non-invasive proteins were found to be closely associated with lung pathology: HP, LTA4H, S100A9, SAA1, SAA2, and SERPINA3. The diagnostic and prognostic value of the identified proteins was verified through the analysis of proteomics data from the plasma of ARDS patients with COVID-19. The results offer possible targets for the early detection and management of direct ARDS/ALI, at least in hyperinflammatory subphenotype.

## Data availability statement

The datasets presented in this study can be found in online repositories. The names of the repository/repositories and accession number(s) can be found below: PXD039618 (ProteomeXchange).

## Ethics statement

The animal study was reviewed and approved by The animal ethics committee of Wuhan Xianran Biotechnology Co., Ltd. (BSMS2022-10-07A).

## Author contributions

Conceptualization, DZ and YS. Data curation, RG. Formal analysis, HL and XZ. Investigation, RG, GL and HL. Methodology, RG. Project administration, RG. Supervision, YS, DZ and CH. Writing—original draft, RG and GL. Writing—review and editing, YS, RG, and JX. All authors have read and agreed to the final version of the manuscript.

## References

[1] Abe T Madotto F Pham T Nagata I Uchida M Tamiya N. Epidemiology and patterns of tracheostomy practice in patients with acute respiratory distress syndrome in icus across 50 countries. Crit Care (London England) (2018) 22(1):195. doi: 10.1186/s13054-018-2126-6 PMC6097245 30115127

[2] Ware LB Matthay MA. The acute respiratory distress syndrome. New Engl J Med (2000) 342(18):1334–49. doi: 10.1056/nejm200005043421806 10793167

[3] Ferguson ND Fan E Camporota L Antonelli M Anzueto A Beale R. The Berlin definition of Ards: an expanded rationale, justification, and supplementary material. Intensive Care Med (2012) 38(10):1573–82. doi: 10.1007/s00134-012-2682-1 22926653

[4] Wilson JG Calfee CS. Ards Subphenotypes: understanding a heterogeneous syndrome. Crit Care (London England) (2020) 24(1):102. doi: 10.1186/s13054-020-2778-x PMC7092435 32204722

[5] Liu C Xiao K Xie L. Advances in the regulation of macrophage polarization by mesenchymal stem cells and implications for Ali/Ards treatment. Front Immunol (2022) 13:928134. doi: 10.3389/fimmu.2022.928134 35880175 PMC9307903

[6] Gillet LC Navarro P Tate S Röst H Selevsek N Reiter L. Targeted data extraction of the Ms/Ms spectra generated by data-independent acquisition: a new concept for consistent and accurate proteome analysis. Mol Cell Proteom: MCP (2012) 11(6):O111.016717. doi: 10.1074/mcp.O111.016717 PMC3433915 22261725

[7] Ludwig C Gillet L Rosenberger G Amon S Collins BC Aebersold R. Data-independent acquisition-based swath-Ms for quantitative proteomics: a tutorial. Mol Syst Biol (2018) 14(8):e8126. doi: 10.15252/msb.20178126 30104418 PMC6088389

[8] Jia B Zhao X Wu D Dong Z Chi Y Zhao J. Identification of serum biomarkers to predict Pemetrexed/Platinum chemotherapy efficacy for advanced lung adenocarcinoma patients by data-independent acquisition (Dia) mass spectrometry analysis with parallel reaction monitoring (Prm) verification. Trans Lung Cancer Res (2021) 10(2):981–94. doi: 10.21037/tlcr-21-153 PMC7947410 33718037

[9] Zhang L Wu F Fan C Huang S Ma Y Chen S. Quantitative phosphoproteomic analysis of mice with liver fibrosis by dia mass spectrometry analysis with prm verification. J Proteomics (2023) 271:104768. doi: 10.1016/j.jprot.2022.104768 36336261

[10] Sun X Li Q Wang J Ma Y Wang M Qin W. Urinary proteome analysis of global cerebral ischemia-reperfusion injury rat model *Via* data-independent acquisition and parallel reaction monitoring proteomics. J Mol Neurosci (2022) 72(9):2020–9. doi: 10.1007/s12031-022-02055-1 PMC9392715 35920976

[11] Rocco PRM Nieman GF. Ards: What experimental models have taught us. Intensive Care Med (2016) 42(5):806–10. doi: 10.1007/s00134-016-4268-9 26928038

[12] Wang Y Wang X Li Y Xue Z Shao R Li L. Xuanfei baidu decoction reduces acute lung injury by regulating infiltration of neutrophils and macrophages *Via* pd-1/Il17a pathway. Pharmacol Res (2022) 176:106083. doi: 10.1016/j.phrs.2022.106083 35033647 PMC8757644

[13] Amatullah H Maron-Gutierrez T Shan Y Gupta S Tsoporis JN Varkouhi AK. Protective function of dj-1/Park7 in lipopolysaccharide and ventilator-induced acute lung injury. Redox Biol (2021) 38:101796. doi: 10.1016/j.redox.2020.101796 33246293 PMC7695876

[14] Ning L Wei W Wenyang J Rui X Qing G. Cytosolic DNA-Sting-Nlrp3 axis is involved in murine acute lung injury induced by lipopolysaccharide. Clin Trans Med (2020) 10(7):e228. doi: 10.1002/ctm2.228 PMC7668192 33252860

[15] Shu T Ning W Wu D Xu J Han Q Huang M. Plasma proteomics identify biomarkers and pathogenesis of covid-19. Immunity (2020) 53(5):1108–22.e5. doi: 10.1016/j.immuni.2020.10.008 33128875 PMC7574896

[16] Qiu Y Wu D Ning W Xu J Shu T Huang M. Post-mortem tissue proteomics reveals the pathogenesis of multi-organ injuries of covid-19. Natl Sci Rev (2021) 8(11):nwab143. doi: 10.1093/nsr/nwab143 34876996 PMC8644997

[17] D’Alessio FR Craig JM Singer BD Files DC Mock JR Garibaldi BT. Enhanced resolution of experimental Ards through il-4-Mediated lung macrophage reprogramming. Am J Physiol Lung Cell Mol Physiol (2016) 310(8):L733–46. doi: 10.1152/ajplung.00419.2015 PMC4836113 26895644

[18] Matute-Bello G Downey G Moore BB Groshong SD Matthay MA Slutsky AS. An official American thoracic society workshop report: features and measurements of experimental acute lung injury in animals. Am J Respir Cell Mol Biol (2011) 44(5):725–38. doi: 10.1165/rcmb.2009-0210ST PMC7328339 21531958

[19] Calfee CS Janz DR Bernard GR May AK Kangelaris KN Matthay MA. Distinct molecular phenotypes of direct vs indirect Ards in single-center and multicenter studies. Chest (2015) 147(6):1539–48. doi: 10.1378/chest.14-2454 PMC4451708 26033126

[20] Peukert K Fox M Schulz S Feuerborn C Frede S Putensen C. Inhibition of caspase-1 with tetracycline ameliorates acute lung injury. Am J Respir Crit Care Med (2021) 204(1):53–63. doi: 10.1164/rccm.202005-1916OC 33760701 PMC8437127

[21] Calfee CS Delucchi K Parsons PE Thompson BT Ware LB Matthay MA. Subphenotypes in acute respiratory distress syndrome: latent class analysis of data from two randomised controlled trials. Lancet Respir Med (2014) 2(8):611–20. doi: 10.1016/s2213-2600(14)70097-9 PMC4154544 24853585

[22] Famous KR Delucchi K Ware LB Kangelaris KN Liu KD Thompson BT. Acute respiratory distress syndrome subphenotypes respond differently to randomized fluid management strategy. Am J Respir Crit Care Med (2017) 195(3):331–8. doi: 10.1164/rccm.201603-0645OC PMC5328179 27513822

[23] Maddali MV Churpek M Pham T Rezoagli E Zhuo H Zhao W. Validation and utility of Ards subphenotypes identified by machine-learning models using clinical data: an observational, multicohort, retrospective analysis. Lancet Respir Med (2022) 10(4):367–77. doi: 10.1016/s2213-2600(21)00461-6 PMC8976729 35026177

[24] Andersen CBF Stødkilde K Sæderup KL Kuhlee A Raunser S Graversen JH. Haptoglobin. Antioxid Redox Signaling (2017) 26(14):814–31. doi: 10.1089/ars.2016.6793 27650279

[25] Kalenka A Feldmann RE Jr. Otero K Maurer MH Waschke KF Fiedler F. Changes in the serum proteome of patients with sepsis and septic shock. Anesth Analgesia (2006) 103(6):1522–6. doi: 10.1213/01.ane.0000242533.59457.70 17122233

[26] Chavez-Bueno S Beasley JA Goldbeck JM Bright BC Morton DJ Whitby PW. ‘Haptoglobin concentrations in preterm and term newborns’. J Perinatol: Off J California Perinatal Assoc (2011) 31(7):500–3. doi: 10.1038/jp.2010.197 21252963

[27] Bowler RP Reisdorph N Reisdorph R Abraham E. Alterations in the human lung proteome with lipopolysaccharide. BMC Pulmonary Med (2009) 9:20. doi: 10.1186/1471-2466-9-20 19432985 PMC2694759

[28] Janz DR Bastarache JA Sills G Wickersham N May AK Bernard GR. Association between haptoglobin, hemopexin and mortality in adults with sepsis. Crit Care (London England) (2013) 17(6):R272. doi: 10.1186/cc13108 PMC4056258 24225252

[29] Larsen R Gozzelino R Jeney V Tokaji L Bozza FA Japiassú AM. A central role for free heme in the pathogenesis of severe sepsis. Sci Trans Med (2010) 2(51):51ra71. doi: 10.1126/scitranslmed.3001118 20881280

[30] Arredouani MS Kasran A Vanoirbeek JA Berger FG Baumann H Ceuppens JL. Haptoglobin dampens endotoxin-induced inflammatory effects both in vitro and in vivo. Immunology (2005) 114(2):263–71. doi: 10.1111/j.1365-2567.2004.02071.x PMC1782073 15667571

[31] Yang F Haile DJ Berger FG Herbert DC Van Beveren E Ghio AJ. Haptoglobin reduces lung injury associated with exposure to blood. Am J Physiol Lung Cell Mol Physiol (2003) 284(2):L402–9. doi: 10.1152/ajplung.00115.2002 12388365

[32] Kubota K Egi M Mizobuchi S. Haptoglobin administration in cardiovascular surgery patients: its association with the risk of postoperative acute kidney injury. Anesth Analgesia (2017) 124(6):1771–6. doi: 10.1213/ane.0000000000002093 28525506

[33] He R Chen Y Cai Q. The role of the Ltb4-Blt1 axis in health and disease. Pharmacol Res (2020) 158:104857. doi: 10.1016/j.phrs.2020.104857 32439596

[34] Xiao Q Dong N Yao X Wu D Lu Y Mao F. Bufexamac ameliorates lps-induced acute lung injury in mice by targeting Lta4h. Sci Rep (2016) 6:25298. doi: 10.1038/srep25298 27126280 PMC4850449

[35] Lee KH Ali NF Lee SH Zhang Z Burdick M Beaulac ZJ. Substrate-dependent modulation of the leukotriene a(4) hydrolase aminopeptidase activity and effect in a murine model of acute lung inflammation. Sci Rep (2022) 12(1):9443. doi: 10.1038/s41598-022-13238-6 35676292 PMC9177663

[36] Wang S Song R Wang Z Jing Z Wang S Ma J. S100a8/A9 in inflammation. Front Immunol (2018) 9:1298. doi: 10.3389/fimmu.2018.01298 29942307 PMC6004386

[37] Vogl T Tenbrock K Ludwig S Leukert N Ehrhardt C van Zoelen MA. Mrp8 and Mrp14 are endogenous activators of toll-like receptor 4, promoting lethal, endotoxin-induced shock. Nat Med (2007) 13(9):1042–9. doi: 10.1038/nm1638 17767165

[38] Boyd JH Kan B Roberts H Wang Y Walley KR. S100a8 and S100a9 mediate endotoxin-induced cardiomyocyte dysfunction *Via* the receptor for advanced glycation end products. Circ Res (2008) 102(10):1239–46. doi: 10.1161/circresaha.107.167544 18403730

[39] Zhao B Lu R Chen J Xie M Zhao X Kong L. S100a9 blockade prevents lipopolysaccharide-induced lung injury *Via* suppressing the Nlrp3 pathway. Respir Res (2021) 22(1):45. doi: 10.1186/s12931-021-01641-y 33549095 PMC7866705

[40] Fontaine M Pachot A Larue A Mougin B Landelle C Venet F. Delayed increase of S100a9 messenger rna predicts hospital-acquired infection after septic shock. Crit Care Med (2011) 39(12):2684–90. doi: 10.1097/CCM.0b013e3182282a40 21765347

[41] Hiroshima Y Hsu K Tedla N Wong SW Chow S Kawaguchi N. S100a8/A9 and S100a9 reduce acute lung injury. Immunol Cell Biol (2017) 95(5):461–72. doi: 10.1038/icb.2017.2 PMC5454315 28074060

[42] Sack GH Jr. Serum amyloid a (Saa) proteins. Sub-cell Biochem (2020) 94:421–36. doi: 10.1007/978-3-030-41769-7_17 32189310

[43] Sack GH Jr. Serum amyloid a - a review. Mol Med (2018) 24(1):46. doi: 10.1186/s10020-018-0047-0 30165816 PMC6117975

[44] Linke RP Meinel A Chalcroft JP Urieli-Shoval S. Serum amyloid a (Saa) treatment enhances the recovery of aggravated polymicrobial sepsis in mice, whereas blocking saa’s invariant peptide results in early death. Off J Int Soc Amyloidosis (2017) 24(sup1):149–50. doi: 10.1080/13506129.2017.1295950 28434296

[45] Cheng N Liang Y Du X Ye RD. Serum amyloid a promotes LPS clearance and suppresses LPS-induced inflammation and tissue injury. EMBO Rep (2018) 19(10):e45517. doi: 10.15252/embr.201745517 30126923 PMC6172460

[46] Cichy J Potempa J Chawla RK Travis J. Stimulatory effect of inflammatory cytokines on alpha 1-antichymotrypsin expression in human lung-derived epithelial cells. J Clin Invest (1995) 95(6):2729–33. doi: 10.1172/jci117975 PMC295956 7769112

[47] Baker C Belbin O Kalsheker N Morgan K. Serpina3 (Aka alpha-1-Antichymotrypsin). Front Biosci (2007) 12:2821–35. doi: 10.2741/2275 17485262

[48] Wiedow O Meyer-Hoffert U. Neutrophil serine proteases: potential key regulators of cell signalling during inflammation. J Internal Med (2005) 257(4):319–28. doi: 10.1111/j.1365-2796.2005.01476.x 15788001

[49] Beatty K Bieth J Travis J. Kinetics of association of serine proteinases with native and oxidized alpha-1-Proteinase inhibitor and alpha-1-Antichymotrypsin. J Biol Chem (1980) 255(9):3931–4. doi: 10.1016/S0021-9258(19)85615-6 6989830

[50] DeCoux A Tian Y DeLeon-Pennell KY Nguyen NT de Castro Brás LE Flynn ER. Plasma glycoproteomics reveals sepsis outcomes linked to distinct proteins in common pathways. Crit Care Med (2015) 43(10):2049–58. doi: 10.1097/ccm.0000000000001134 PMC4573827 26086942

[51] Čaval T Lin YH Varkila M Reiding KR Bonten MJM Cremer OL. Glycoproteoform profiles of individual patients’ plasma alpha-1-Antichymotrypsin are unique and extensively remodeled following a septic episode. Front Immunol (2020) 11:608466. doi: 10.3389/fimmu.2020.608466 33519818 PMC7840657

[52] Wang X Chen B Chen C. Identification of biomarkers and candidate small-molecule drugs in lipopolysaccharide (Lps)-induced acute lung injury by bioinformatics analysis. Allergol Immunopathol (2023) 51(1):44–53. doi: 10.15586/aei.v51i1.755 36617821

[53] O’Donovan DA Kelly CJ Bouchier-Hayes DM Grace P Redmond HP Burke PE. Alpha-1-Antichymotrypsin is an effective inhibitor of pancreatitis-induced lung injury. Eur J Gastroenterol Hepatol (1995) 7(9):847–52. 8574716

[54] D’Alessandro A Thomas T Dzieciatkowska M Hill RC Francis RO Hudson KE. Serum proteomics in covid-19 patients: altered coagulation and complement status as a function of il-6 level. J Proteome Res (2020) 19(11):4417–27. doi: 10.1021/acs.jproteome.0c00365 PMC7640953 32786691

[55] Geyer PE Arend FM Doll S Louiset ML Virreira Winter S Müller-Reif JB. High-resolution serum proteome trajectories in covid-19 reveal patient-specific seroconversion. EMBO Mol Med (2021) 13(8):e14167. doi: 10.15252/emmm.202114167 34232570 PMC8687121

[56] Mohammed Y Goodlett DR Cheng MP Vinh DC Lee TC McGeer A. Longitudinal plasma proteomics analysis reveals novel candidate biomarkers in acute covid-19. J Proteome Res (2022) 21(4):975–92. doi: 10.1021/acs.jproteome.1c00863 35143212

[57] Dong X Zhu Z Wei Y Ngo D Zhang R Du M. Plasma insulin-like growth factor binding protein 7 contributes causally to Ards 28-day mortality: evidence from multistage mendelian randomization. Chest (2021) 159(3):1007–18. doi: 10.1016/j.chest.2020.10.074 PMC8501007 33189655

[58] Yuan Z Wang T Wen F. Itraq-based proteomic analysis reveals mitochondrial’damage’-associated molecular patterns are involved in pulmonary inflammation in lypopolysaccharide-induced acute lung injury. Eur Respir Soc (2018) 52(suppl 62):PA4289. doi: 10.1183/13993003.congress-2018.PA4289

[59] Battaglini D Al-Husinat L Normando AG Leme AP Franchini K Morales M. Personalized medicine using omics approaches in acute respiratory distress syndrome to identify biological phenotypes. Respir Res (2022) 23(1):318. doi: 10.1186/s12931-022-02233-0 36403043 PMC9675217

