# Supplementary material for: Integrative proteomic profiling of lung tissues and blood in acute respiratory distress syndrome
Source: Front Immunol. 2023 May 1;14:1158951. doi: 10.3389/fimmu.2023.1158951 (PMC10184823; doi:10.3389/fimmu.2023.1158951)
Supplement: Supplementary file 1 [file DataSheet_1.zip › Data Sheet 1/Supplemental Table 3. PRM validation in lung samples.docx]

**Table 3.** DEPs from mice lung validated by PRM.

| **Accession** | **Gene Name** | **Protein Description** | **Proteomics** | | **PRM Validation** | |
| --- | --- | --- | --- | --- | --- | --- |
|  |  |  | **LPS/Control** | **P_Value** | **LPS/Control** | **P_Value** |
| O08692 | Ngp | Neutrophilic granule protein  OS=Mus musculus OX=10090  GN=Ngp PE=1 SV=1 | 11.692007 | 8.06E-08 | 13.56408733 | 3.02897E-05 |
| O35744 | Chil3 | Chitinase-like protein 3  OS=Mus musculus OX=10090  GN=Chil3 PE=1 SV=2 | 3.958488 | 6.24E-06 | 6.653173626 | 6.08135E-05 |
| P10810 | Cd14 | Monocyte differentiation antigen CD14  OS=Mus musculus OX=10090  GN=Cd14 PE=1 SV=1 | 10.07505315 | 1.41335E-06 | 16.10407581 | 0.000182739 |
| P11672 | Lcn2 | Neutrophil gelatinase-associated lipocalin  OS=Mus musculus OX=10090  GN=Lcn2 PE=1 SV=1 | 16.43866174 | 1.2903E-09 | 26.84251615 | 1.68968E-05 |
| P14901 | Hmox1 | Heme oxygenase 1 OS=Mus musculus  OX=10090 GN=Hmox1 PE=1 SV=1 | 4.566065898 | 1.24068E-06 | 9.741409315 | 9.0797E-06 |
| P24527 | Lta4h | Leukotriene A-4 hydrolase OS=Mus musculus OX=10090 GN=Lta4h PE=1 SV=4 | 1.503531347 | 5.35468E-05 | 1.842505307 | 0.00023042 |
| P31725 | S100a9 | Protein S100-A9 OS=Mus musculus  OX=10090 GN=S100a9 PE=1 SV=3 | 7.306475507 | 4.6663E-08 | 16.7017871 | 4.65391E-05 |
| P33622 | Apoc3 | Apolipoprotein C-III OS=Mus musculus  OX=10090 GN=Apoc3 PE=1 SV=2 | 0.632442957 | 0.014539453 | 0.445280356 | 0.045944663 |
| P55065 | Pltp | Phospholipid transfer protein  OS=Mus musculus OX=10090  GN=Pltp PE=1 SV=1 | 0.598471067 | 0.000510544 | 0.536510643 | 0.003049559 |
| Q61096 | Prtn3 | Myeloblastin OS=Mus musculus  OX=10090 GN=Prtn3 PE=1 SV=2 | 12.12974583 | 6.29725E-07 | 3.728583159 | 0.000947543 |
| Q61646 | Hp | Haptoglobin OS=Mus musculus  OX=10090 GN=Hp PE=1 SV=1 | 2.56252842 | 0.030826131 | 3.244998585 | 0.04159417 |
| P04918 | Saa3 | Serum amyloid A-3 protein  OS=Mus musculus OX=10090  GN=Saa3 PE=1 SV=1 | 81.14207311 | 1.18602E-05 | 736.3847718 | 0.000434289 |
| P05366 | Saa1 | Serum amyloid A-1 protein OS=Mus musculus OX=10090 GN=Saa1 PE=1 SV=2 | 61.56986882 | 2.05909E-06 | 472.7549671 | 0.000133591 |
