# Supplementary material for: Integrative proteomic profiling of lung tissues and blood in acute respiratory distress syndrome
Source: Front Immunol. 2023 May 1;14:1158951. doi: 10.3389/fimmu.2023.1158951 (PMC10184823; doi:10.3389/fimmu.2023.1158951)
Supplement: Supplementary file 1 [file DataSheet_1.zip › Data Sheet 1/Supplemental Table 4. PRM validation in lung samples.docx]

**Table 4.** DEPs from mice serumg validated by PRM.

| **Accession** | **Gene Name** | **Protein Description** | **Proteomics** | | **PRM Validation** | |
| --- | --- | --- | --- | --- | --- | --- |
|  |  |  | **LPS/Control** | **P_Value** | **LPS/Control** | **P_Value** |
| O08692 | Ngp | Neutrophilic granule protein  OS=Mus musculus OX=10090  GN=Ngp PE=1 SV=1 | 5.819534412 | 0.001095147 | 3.866139341 | 0.018516449 |
| O08742 | Gp5 | Platelet glycoprotein V  OS=Mus musculus OX=10090  GN=Gp5 PE=1 SV=1 | 0.487120048 | 0.032347644 | 0.395405155 | 0.01518027 |
| O35744 | Chil3 | Chitinase-like protein 3  OS=Mus musculus OX=10090  GN=Chil3 PE=1 SV=2 | 5.551148494 | 0.000597194 | 9.322412729 | 0.008748684 |
| P10810 | Cd14 | Monocyte differentiation antigen CD14  OS=Mus musculus OX=10090  GN=Cd14 PE=1 SV=1 | 11.60476217 | 1.44378E-06 | 140.9824664 | 0.001936232 |
| P11672 | Lcn2 | Neutrophil gelatinase-associated lipocalin  OS=Mus musculus OX=10090  GN=Lcn2 PE=1 SV=1 | 35.7013981 | 9.54168E-08 | 48.27441014 | 7.78325E-05 |
| P33622 | Apoc3 | Apolipoprotein C-III OS=Mus musculus  OX=10090 GN=Apoc3 PE=1 SV=2 | 0.597453974 | 0.002173896 | 0.403960551 | 0.001790541 |
| P48759 | Ptx3 | Pentraxin-related protein PTX3  OS=Mus musculus OX=10090  GN=Ptx3 PE=1 SV=2 | 19.18872444 | 9.84611E-06 | 13.07231778 | 0.014157809 |
| P55065 | Pltp | Phospholipid transfer protein  OS=Mus musculus OX=10090  GN=Pltp PE=1 SV=1 | 0.575822802 | 0.000850417 | 0.367317418 | 0.000957999 |
| Q02105 | C1qc | Complement C1q subcomponent subunit C  OS=Mus musculus OX=10090  GN=C1qc PE=1 SV=2 | 0.47127564 | 0.024141049 | 0.371829288 | 0.021343835 |
| Q61646 | Hp | Haptoglobin OS=Mus musculus  OX=10090 GN=Hp PE=1 SV=1 | 11.16890522 | 0.008415072 | 34.97105602 | 0.004226621 |
| P05366 | Saa1 | Serum amyloid A-1 protein  OS=Mus musculus OX=10090  GN=Saa1 PE=1 SV=2 | 75.43535753 | 2.67853E-07 | 6896.109755 | 3.66741E-06 |
| P05367 | Saa2 | Serum amyloid A-2 protein  OS=Mus musculus OX=10090  GN=Saa2 PE=1 SV=1 | 733.3882395 | 4.38802E-08 | 792.1978923 | 1.46869E-05 |
| Q3V2T4 | Krtdap | Keratinocyte differentiation-associated protein OS=Mus musculus OX=10090  GN=Krtdap PE=2 SV=1 | 0.384742272 | 1.84476E-05 | 0.212485135 | 0.007833193 |
