# Supplementary material for: Integrative proteomic profiling of lung tissues and blood in acute respiratory distress syndrome
Source: Front Immunol. 2023 May 1;14:1158951. doi: 10.3389/fimmu.2023.1158951 (PMC10184823; doi:10.3389/fimmu.2023.1158951)
Supplement: Supplementary file 1 [file DataSheet_1.zip › Data Sheet 1/Supplementary Figures.pdf]

Supplementary Material

Integrative Proteomic Profiling of Lung Tissues and Blood in Acute Respiratory Distress Syndrome

Rui Gong[1], Hong Luo[2], Gangyu Long[2], Jiqian Xu[3], Chaolin Huang[2], Xin Zhou[4], You Shang[1,3,*], Dingyu Zhang[1-3,*]

Affiliation
[1] ~~The First Affiliated Hospital of USTC, Division of Life Sciences and Medicine, University of Science and Technology of China, Hefei, Anhui, China~~
[2] ~~Wuhan Jinyintan Hospital, Tongji Medical College, Huazhong University of Science and Technology (HUST), Wuhan, Hubei, China~~
[3] ~~SpecAlly Life Technology Co., Ltd, Wuhan, Hubei, China~~

* **Correspondence:**
Dingyu Zhang
Zhangdingyu2021@126.com;

You Shang
you_shanghust@163.com;

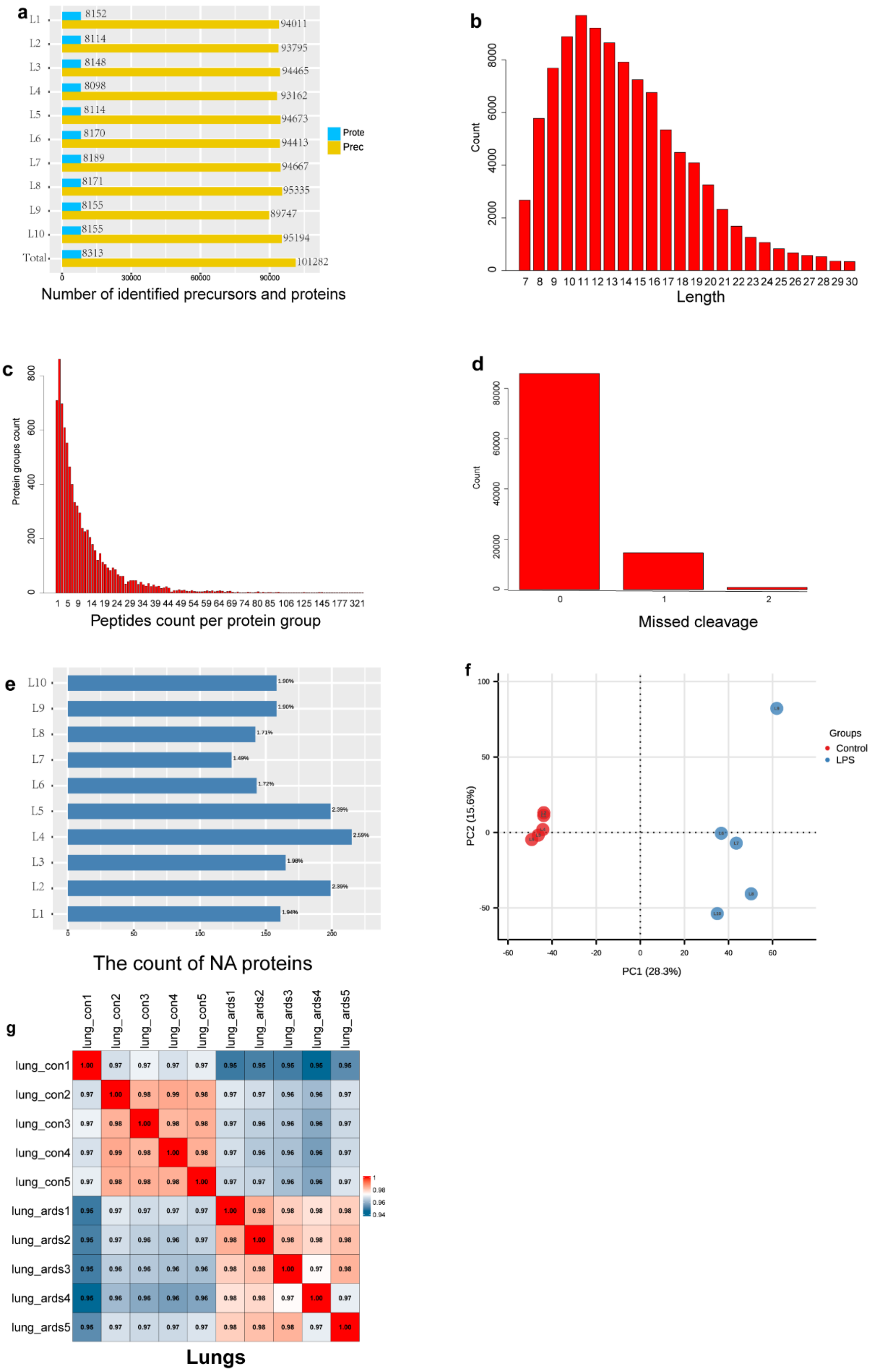

a
L1
L2
L3
L4
L5
L6
L7
L8
L9
L10
Total
8152
94011
8114
93795
8148
94465
8098
93162
8114
94673
8170
94413
8189
94667
8171
95335
8155
89747
8155
95194
8313
101282
Prote
Prec
0
30000
60000
90000
Number of identified precursors and proteins
b
Count
0
2000
4000
6000
8000
7 8 9 10 11 12 13 14 15 16 17 18 19 20 21 22 23 24 25 26 27 28 29 30
Length
c
Protein groups count
0
200
400
600
800
1 5 9 14 19 24 29 34 39 44 49 54 59 64 69 74 80 85 106 125 145 177 321
Peptides count per protein group
d
Count
0
20000
40000
60000
80000
0
1
2
Missed cleavage
e
L10
L9
L8
L7
L6
L5
L4
L3
L2
L1
0
50
100
150
200
The count of NA proteins
f
PC2 (15.6%)
100
50
0
-50
-60
-40
-20
0
20
40
60
PC1 (28.3%)
Groups
Control
LPS
g
lung_con1
lung_con2
lung_con3
lung_con4
lung_con5
lung_ards1
lung_ards2
lung_ards3
lung_ards4
lung_ards5
1
0.98
0.96
0.94
Lungs

Figure S1. Data Filtering and Data Quality assessing in lung samples. (a) The identified proteins and precursors in lung samples; (b) Peptide length distribution; (c) Peptide number distribution; (d) Missed cleavage sites distribution; (e) Missing data distribution; (f) Principal Component Analysis (PCA); (g) Pearson's correlation analysis.

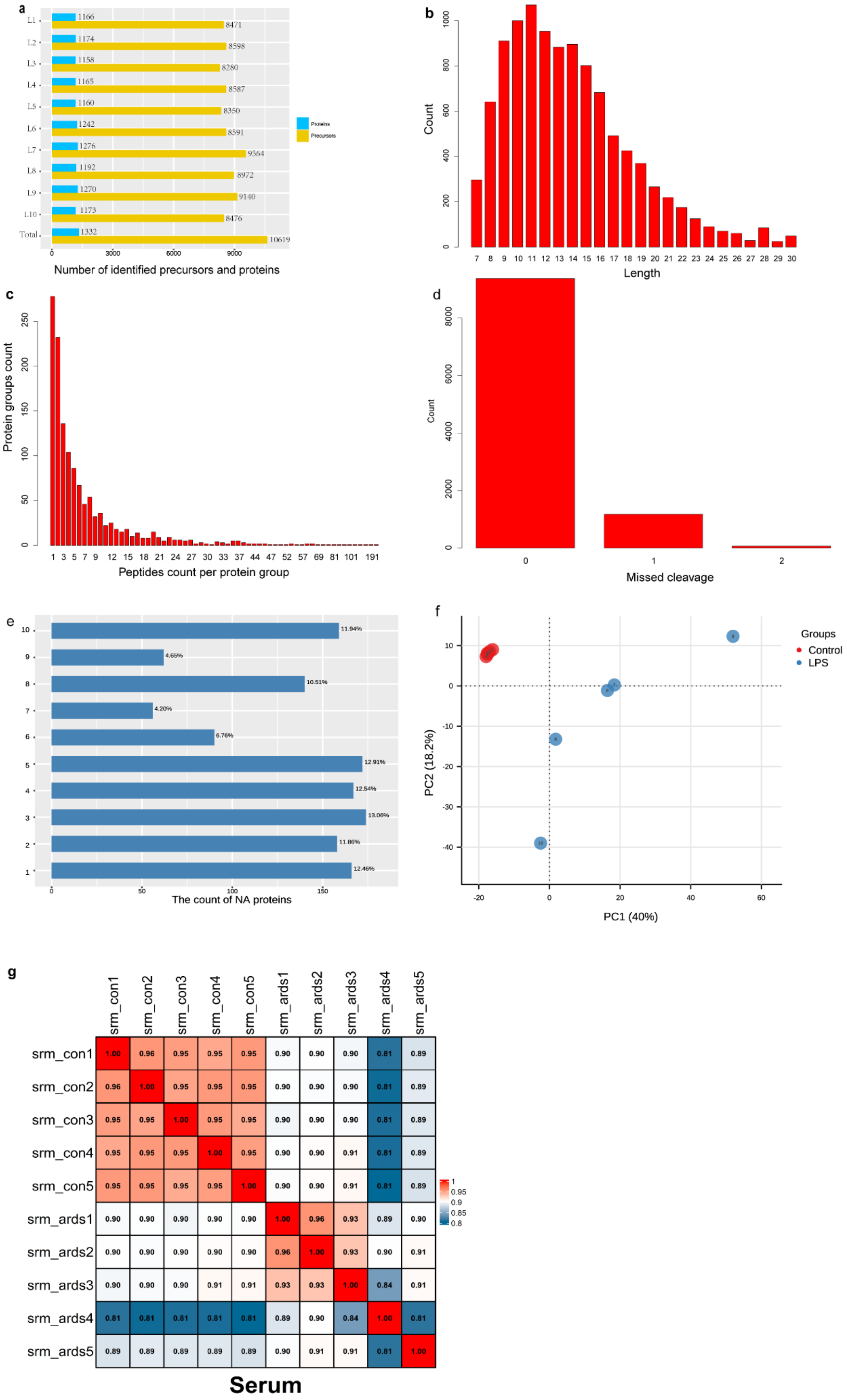
a
L1 1166 8471
L2 1174 8598
L3 1158 8280
L4 1165 8587
L5 1160 8350
L6 1242 8591
L7 1276 9564
L8 1192 8972
L9 1270 9140
L10 1173 8476
Total 1332 10619
Proteins
Precursors
Number of identified precursors and proteins
b
Count
Length
c
Protein groups count
Peptides count per protein group
d
Count
Missed cleavage
e
11.94%
4.65%
10.51%
4.20%
6.76%
12.91%
12.54%
13.06%
11.86%
12.46%
The count of NA proteins
f
Groups
Control
LPS
PC2 (18.2%)
PC1 (40%)
g
srm_con1 1.00 0.96 0.95 0.95 0.95 0.90 0.90 0.90 0.81 0.89
srm_con2 0.96 1.00 0.95 0.95 0.95 0.90 0.90 0.90 0.81 0.89
srm_con3 0.95 0.95 1.00 0.95 0.95 0.90 0.90 0.90 0.81 0.89
srm_con4 0.95 0.95 0.95 1.00 0.95 0.90 0.90 0.91 0.81 0.89
srm_con5 0.95 0.95 0.95 0.95 1.00 0.90 0.90 0.91 0.81 0.89
srm_ards1 0.90 0.90 0.90 0.90 0.90 1.00 0.96 0.93 0.89 0.90
srm_ards2 0.90 0.90 0.90 0.90 0.90 0.96 1.00 0.93 0.90 0.91
srm_ards3 0.90 0.90 0.90 0.91 0.91 0.93 0.93 1.00 0.84 0.91
srm_ards4 0.81 0.81 0.81 0.81 0.81 0.89 0.90 0.84 1.00 0.81
srm_ards5 0.89 0.89 0.89 0.89 0.89 0.90 0.91 0.91 0.81 1.00
Serum

Figure S2. Data Filtering and Data Quality assessing in serum samples. (a) The identified proteins and precursors in serum samples; (b) Peptide length distribution; (c) Peptide number distribution; (d) Missed cleavage sites distribution; (e) Missing data distribution; (f) Principal Component Analysis (PCA); (g) Pearson's correlation analysis.
